# Manipulations of Wavefront Propagation: Useful Methods and Applications for Interferometric Measurements and Scanning

**DOI:** 10.1155/2017/7293905

**Published:** 2017-08-21

**Authors:** Avi Karsenty, Eitan Novoselski, Ariel Yifrach, Emmanuel Lanzmann, Yoel Arieli

**Affiliations:** ^1^Department of Applied Physics/Electro-Optics Engineering, Faculty of Engineering, Lev Academic Center, 9116001 Jerusalem, Israel; ^2^Shlonsky St. 31, 69400 Tel Aviv, Israel

## Abstract

Phase measurements obtained by high-coherence interferometry are restricted by the 2*π* ambiguity, to height differences smaller than *λ*/2. A further restriction in most interferometric systems is for focusing the system on the measured object. We present two methods that overcome these restrictions. In the first method, different segments of a measured wavefront are digitally propagated and focused locally after measurement. The divergent distances, by which the diverse segments of the wavefront are propagated in order to achieve a focused image, provide enough information so as to resolve the 2*π* ambiguity. The second method employs an interferogram obtained by a spectrum constituting a small number of wavelengths. The magnitude of the interferogram's modulations is utilized to resolve the 2*π* ambiguity. Such methods of wavefront propagation enable several applications such as focusing and resolving the 2*π* ambiguity, as described in the article.

## 1. Introduction

Highly accurate phase measurements are very important in applications of surface measurements [1–3]. Nevertheless, phase measurements obtained by high-coherence interferometry are restricted by the 2*π* ambiguity to height differences smaller than *λ*/2. In order to increase the dynamic range of 3D measurements for continuous objects, known unwrapping algorithms can be used [4, 5]. However, for step-like objects, several different measurements, each consisting of a different wavelength, must be applied [6]. By comparing the phases obtained by these measurements at each point of the object, the 2*π* ambiguity is resolved and a considerably larger dynamic range can be measured. In this article, we suggest to overcome this restriction regarding a step-like object by digitally propagating the measured wavefront reflected by the object and refocusing it. By focusing each segment of the wavefront locally and by retaining the distances by which each part of the wavefront was propagated and focused, the information as to resolve the 2*π* ambiguity is provided. Moreover, this procedure obviates the need for autofocusing mechanism simply by taking a measurement and propagating it to the image plane.

## 2. Model

### 2.1. Gibbs Phenomenon

**Figure 1:**
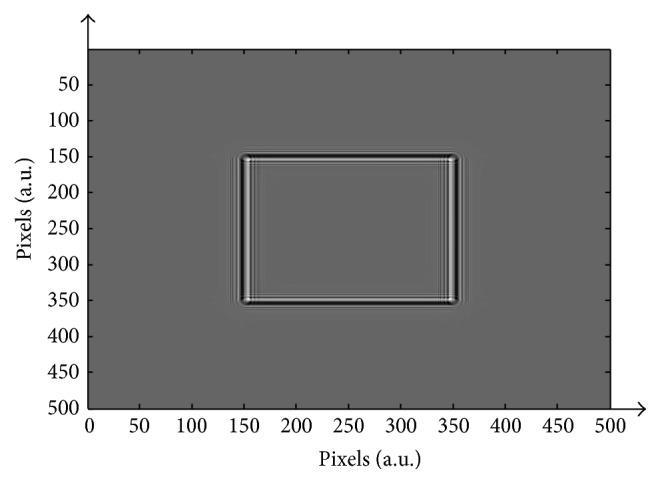
2D grayscale representation of the height levels.

**Figure 2:**
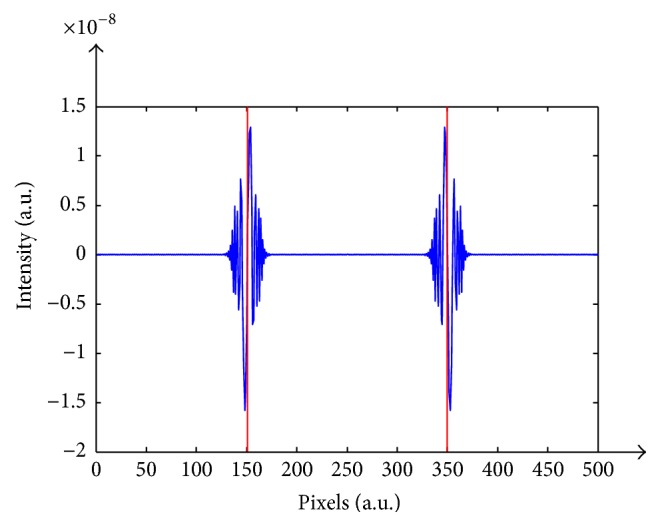
Horizontal intersection of that same paradigm.

The complex amplitude of a wavefront obtained by a uniformly collimated monochromatic beam of light reflected from an object is given by

(1) $$\begin{array}{c} U\left(\;x,y\;\right)=A\left(\;x,y\;\right)\exp\left[\;i\varphi \left(\;x,y\;\right)\;\right], \end{array}$$

where *A* is the reflectance of the object and *φ* is the phase map of the reflected wavefront which is related to the height map *h*(*x*, *y*) of the object. By approximation, the phase map *φ* is given by

(2) $$\begin{array}{c} \varphi \left(\;x,y\;\right)=-\frac{4\pi h\left(x,y\right)}{\lambda \;mod\;2\pi }, \end{array}$$

where *λ* is the wavelength and mod 2*π* is the phase modulus of 2*π*. Accordingly, the height map of the object is restricted by the 2*π* ambiguity, and it can be reconstructed unambiguously from the phase map *φ*, provided that the pixel-to-pixel height differences are less than *λ*/2. Moreover, when focusing the interferometer on a certain plane ringing artifacts occur as a result of unfocused levels which influence focused planes. This effect, which is created due to the propagation of the wavefront and which belongs to the unfocused sections of the measured surface to the same level as the focused sections, is known as the Gibbs phenomenon. The ringing artifacts for a piston object with height differences of 108 *μ*m are illustrated in Figure 1. The figure shows a two-dimensional grayscale representation of the intensities of interferometric measurement of a wavefront of light with *λ* = 0.5 *μ*m reflected from the two height levels.  Figure 2 is a horizontal intersection of the reconstructed heights.

The gray levels and the reconstructed heights, of both the upper and lower segments of the surface, are identical due to the 2*π* ambiguity. Moreover, due to the focusing problem, a distortion of ±15 nm is perceived on the border of the adjoining height levels. It is important to note that unwrapping is inefficient in this case. The variety of potential levels, complying with the condition of *h* = *mλ*/2, makes it impossible to deduce the real deviation.

### 2.2. The Focusing Criteria Package

There are many existing autofocus methods, which are used for various types of optical devices. Most of them are based on sharpness function, which is a real-valued estimate of the image's sharpness [7]. In most of these methods, several images are taken in different planes, and the sharpness function is calculated where the focal plane is defined as the plane for which it reaches a single optimum. As reviewed in [7], the existing sharpness functions are based on several techniques and criteria. Among others, we can find the image derivative [8] and the Fourier Transform [9].

As part of the above presented methods, several “focusing criteria” have been adapted and developed by our team. These criteria define the wavefront of a focused image and are based on the changes in diffraction and Gibbs phenomena when a wavefront is propagated from a plane to another one.

*(i) The Fourier Transform Criterion*. In this criterion [10], the spatial behavior of the wavefront *U*(*x*, *y*) is checked according to the integral of the absolute value of the Fourier Transform of the squared wavefront [11]:

(3) $$\begin{array}{c} C_{FFT}\left(\;U\left(\;x,y\;\right)\;\right)=\iint \left|\;I\left[\;U{\left(\;x,y\;\right)}^{2}\;\right]\;\right|dx\;dy. \end{array}$$

The rationale behind this method is as follows: since in focused image the amplitude and phase changes in the image, as a consequence of the diffraction and Gibbs phenomena, are the smallest, at least locally, the value of the integral will be also the smallest for a focused image.

*(ii) The Standard Deviation Criterion*. This criterion checks the shape of the spatial wavefront behavior *U*(*x*, *y*) as a function of the local standard deviation of the squared wavefront [10]:

(4) $$\begin{array}{c} C_{Std}\left(\;U\left(\;x,y\;\right)\;\right)=\sigma \left[\;U{\left(\;x,y\;\right)}^{2}\;\right], \end{array}$$

where *σ* represents the standard deviation operator. The assumption, on which this criterion is based, is as follows: since, for nonfocused image, the changes in the amplitude in the image are much stronger than for a focused image, the value of the overall standard deviation of the squared wavefront will be the smallest at the focused image.

*(iii) The Gradient Criterion*. Based on this criterion, the focusing level of the image is checked as a function of the local integral of the modulus of the local gradient of the square wavefront [10]:

(5) $$\begin{array}{c} C_{Grad}\left(\;U\left(\;x,y\;\right)\;\right)=\iint \left|\;\vec{grad}\left[\;U{\left(\;x,y\;\right)}^{2}\;\right]\;\right|dx\;dy. \end{array}$$

Since the changes in amplitude and phase in a focused image, as a function of diffraction and Gibbs phenomena [11, 12], are the smallest, the value of the integral will locally remain the smallest for a focused image.

*(iv) The Entropy Criterion*. At the end, the last criterion is based on the previous one and is the local integral of the modulus of the gradient of the complex wavefront. This criterion takes also into account not only the intensity function as the abovementioned methods [8, 9] but also the phase function of the complex amplitude of the image [10]:

(6) $$\begin{array}{c} C_{Ent}\left(\;U\left(\;x,y\;\right)\;\right)=\iint \left\|\;\vec{grad}\left[\;U\left(\;x,y\;\right)\;\right]\;\right\|dx\;dy. \end{array}$$

### 2.3. Beam Propagation

There are two mathematically equivalent formulas which enable the propagation of a wavefront, depending on whether it is preferred to decompose the wavefront as a sum of spherical waves or as a sum of plane waves. For the spherical wave decomposition, the formula of wavefront propagation to a plane *P*_*z*_ located at a distance *z* from the plane *P* positioned at *z* = 0 is [10]

(7) fzx,y=z2πf0∗exp⁡2iπεzRz/λRz22iπλ−1Rz,

where *λ* is the wavelength and

(8) $$\begin{array}{c} R_{z}=\sqrt{x^{2}+y^{2}+z^{2}}, \end{array}$$

*ɛ*(*z*) = ±1 is the sign of *z*, and the symbol *∗* stands for convolution. For the plane waves' decomposition, the formula of wavefront propagation, known also as the “angular spectrum,” is given by [11]

(9) $$\begin{array}{c} f_{z}=F\left\{\;F^{-1}\left(\;f_{0}\;\right)\cdot \exp\left[\;2i\pi z\sqrt{\frac{1}{\lambda^{2}}-\nu_{x}^{2}-\nu_{y}^{2}}\;\right]\;\right\}, \end{array}$$

where *ν*_*x*_ and *ν*_*y*_ are the spatial frequencies and *F* stands for the Fourier Transform. For both (7) and (9), the Fast-Fourier Transform (FFT) algorithm can be implemented and the diffracted wave can therefore be computed very efficiently.

## 3. Results

### 3.1. Refocusing

**Figure 3:**
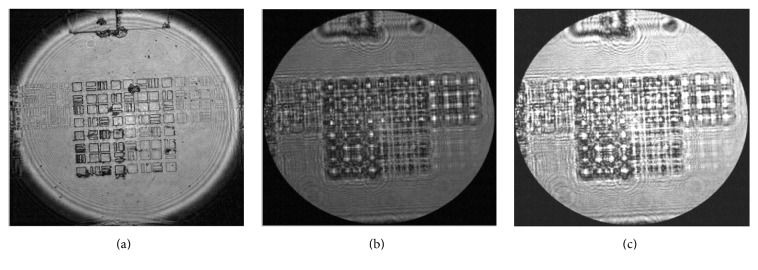
An image of an object illuminated by coherent light. (a) At Image plane, (b) defocus of 400 *μ*m, and (c) defocus of −400 *μ*m.

Figures 3(a), 3(b), and 3(c) show an image of a measured wavefront of an object illuminated by coherent light where in 3(a) the object is located at image plane and in 3(b) and 3(c) the object is, respectively, in defocus of 400 *μ*m and −400 *μ*m. As a result the defocused image is blurred and possesses a greater amount of ripples which occur around edges, relative to the focused image.

### 3.2. Virtual Autofocus

**Figure 4:**
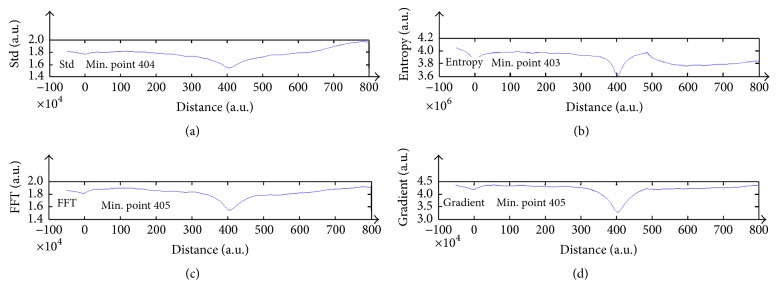
Focus criteria values measured for defocus of 390 *μ*m. (a) Std, (b) entropy, (c) FFT, and (d) gradient.

**Figure 5:**
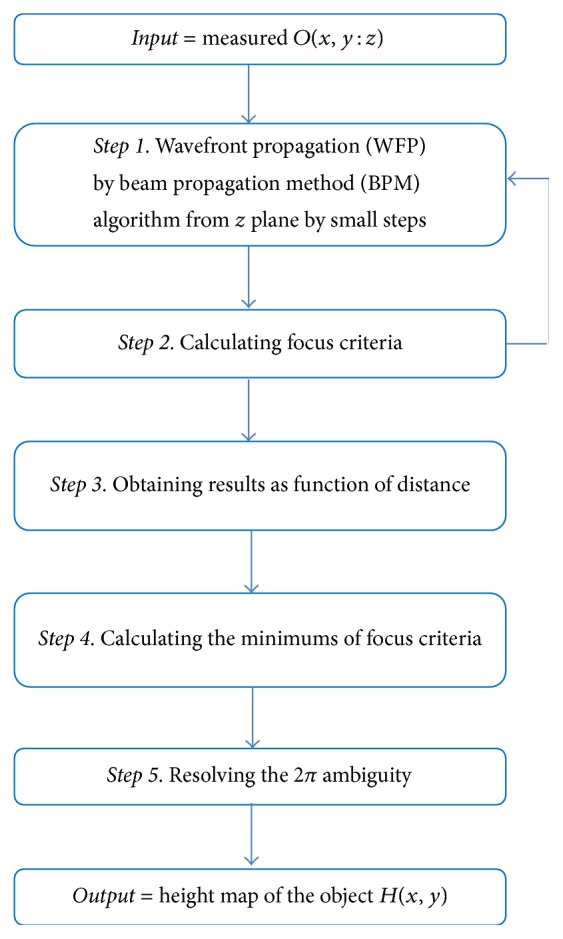
Flow chart of the first method for resolving the 2*π* ambiguity.

The defocused wavefront was propagated by means of BPM program, towards the image plane while calculating the focus criteria values. In Figure 4 we present the calculated focus criteria values for continuous propagating distances. In this figure, we can clearly observe that the criteria values are changing as a function of the wavefront propagation and that the minimum value is near the focal plan which is 400 *μ*m. The added value of such an approach (Figure 5) is that there is no need for any additional mechanical equipment, since it is realizable through the algorithms of the wavefront propagation. Such a technique can be called Virtual Autofocus.

### 3.3. 2*π* Ambiguity

**Figure 6:**
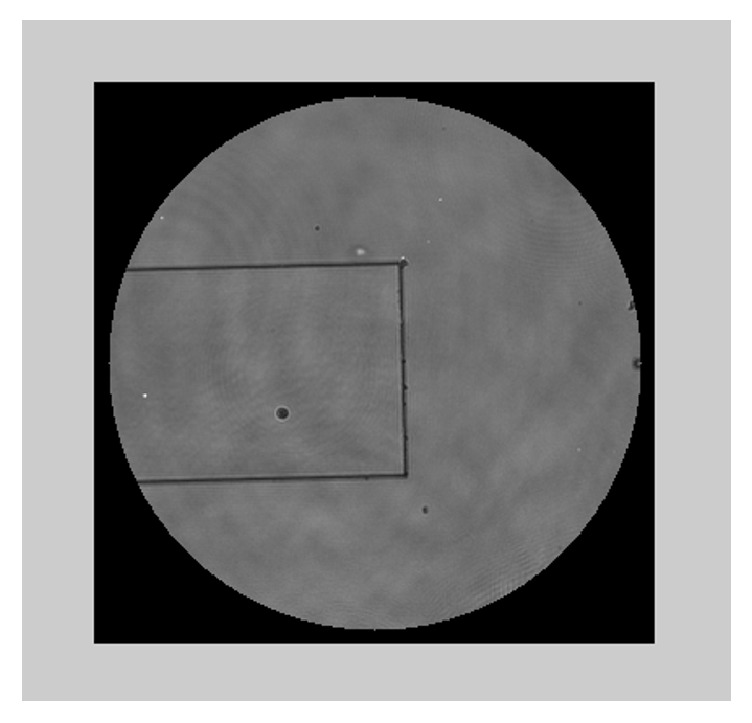
A certified step-height standard of 929 nm height (by VLSI-standards).

This technique can also be utilized for resolving the 2*π* ambiguity when refocusing each segment of the wavefront locally. Once the wavefront has been measured for a certain plane *P*, each segment of the wavefront can be propagated to a certain plane at which the image has a minimum amount of ripples around edges. This plane can be defined as the image plane of that segment. The distance by which each part of the wavefront has been propagated provides the additional information for resolving the 2*π* ambiguity. We have measured the wavefront reflected from an object containing two segments, a substrate and a step height as shown in Figure 6. The object is a chrome-plated certified step-height standard of 929 nm height (by VLSI-standards) which is well beyond the 2*π* ambiguity range. The measurements were taken with an imaging interferometer [3, 11] mounted on a standard Olympus white light microscope, using a magnification of ×100, a NA of 0.9, and a 680 nm wavelength illumination. The different segments of the measured wavefront, the substrate and the step height, were propagated separately to divergent planes. As before, the image of each segment which appeared to include minimum distortions was defined as the focused image of that segment. The difference between the propagation distances to the image plane of the step height and the image plane of the substrate is the height of the step relative to the substrate. The height step that was calculated by the global approach was 896 nm while the height step calculated by the local approach was 912 nm. The variation between these results is within 1.7% from each other and within 1.5% from the nominal height of the object. However, as long as the accuracy of the wavefront propagation result is better than *λ*/2, it can be combined with the interferometric measurements to resolve the 2*π* ambiguity. Accordingly, the interferometric measurement gives the most accurate result, while the digital wavefront propagation provides additional information for the solution of 2*π* ambiguities. It should be noted that this method requires some a priori knowledge about the configuration of the step-like object and a minimal number of pixels within each object's segment in order to obtain an unambiguous result.

### 3.4. A Suggested Method for Resolving the 2*π* Ambiguity

**Figure 7:**
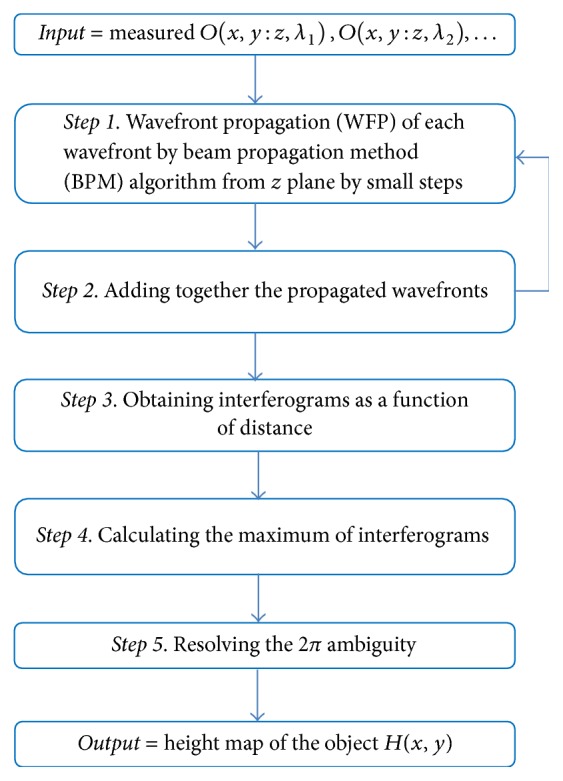
Flow chart of the second method for resolving the 2*π* ambiguity.

Here we suggest another method (Figure 7) for resolving the 2*π* ambiguity by measuring the object using two or more discreet wavelengths.

The object's heights are obtained in the same fashion as in noncoherent interferometry, by adding the interference patterns obtained by the different discreet wavelengths to obtain the interferogram and calculating the position of the centered maximum of the interferogram. The main advantage here is that, as opposed to noncoherent interferometry, there is no need for moving mirror to obtain the interferogram. Since the light for each wavelength is coherent, the measured wavefronts can be propagated electronically. It should be noted that using discreet number of wavelengths the interferogram is periodic and the resolution is limited. However, since this technique may be used only for resolving the 2*π* ambiguity, the resolution is not so important.

**Figure 8:**
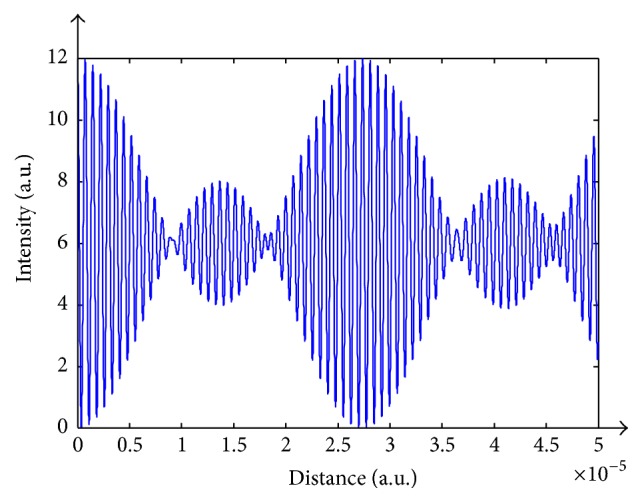
Discrete spectrum interferogram.

**Figure 9:**
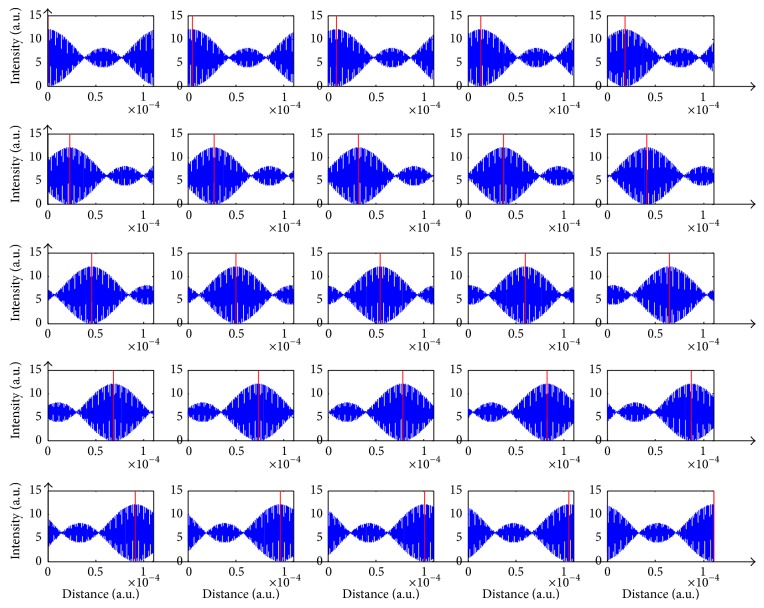
Sequence of interferograms.

We have simulated this technique by carefully selecting three wavelengths that will yield satisfactory results: *λ*_1_ = 720 nm, *λ*_2_ = 740 nm, and *λ*_3_ = 760 nm. An interferogram generated by propagating the three wavefronts is shown in Figure 8. On the other hand, Figure 9 shows a sequence of the discrete spectrum interferograms at divergent levels.

**Figure 10:**
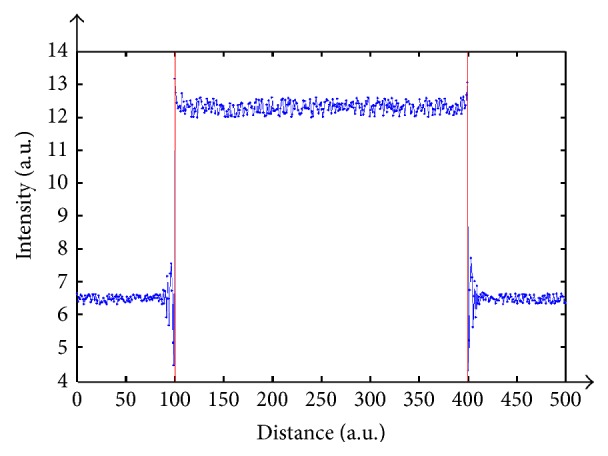
Maxima for both surface sections considering a noised interferogram.

At differing heights of the sample, the same interferogram initializes at distinct positions, allowing the retrieval of sample's shape. As illustrated above, the centered maximum value relocates itself linearly as a function of depth changes. Nevertheless, as perceived from the figure, amplitudes can coincide for two different levels, and, as a consequence, several multiples of the periodic cycle are exactly lying, one on each other. Truly, such confinement can be neglected. As for ranges of 15 to 55 microns it can be detected, depending on the opted wavelengths. For longer wavelengths this range increases, and therefore usage of infrared illumination is indicated. Another criterion influencing the periodic segment is Δ*λ*. A small Δ*λ* is preferable since closer wavelengths generate large periodic segments. We have checked the immunity of this method to noise by adding random noise to the interferograms as shown in Figure 10.

According to our results, even in presence of noise artifacts it is feasible to distinguish the precise position of each height according to the amplitude of intensity modulations. Moreover, it is well known that the usage of multiple wavelengths is sensitive to noise, especially for close wavelengths. Our method is not influenced by such noise since it seeks for a graph's maximum obtained as a result of multiple propagations and for a maxima obtained from the global propagation of each wavefront in each field of vision and not only for local results. So, even if there is any local concern, the global propagation enables its repair.

## 4. Conclusions

In conclusion, we have introduced two new methods in order to resolve the 2*π* ambiguity in interferometric measurements. The first method consists in beam propagation of the measured wavefront. This method enables the extension of the dynamic range of single wavelength interferometry beyond the limitation of *λ*/2 even for step-like objects. The second technique utilizes several wavelengths in order to acquire a discrete wavelengths interferometry measurements as well as obtaining the interferograms simply by electronic propagating and adding the wavefronts without the need for moving the interferometer's mirror. Divergent sections of the interferogram represent differing heights, while each section undergoes different intensity modulations. We have demonstrated and implemented these methods, proving their promising usefulness. In addition to resolving the 2*π* ambiguity, these procedures obviate the need for focusing the interferometer on the measured object.
